# ⁶⁸GA-Pentixafor PET/CT: a non-invasive molecular alternative to adrenal venous sampling in primary aldosteronism

**DOI:** 10.1186/s41824-026-00287-7

**Published:** 2026-04-17

**Authors:** Parth Bambhroliya, Kanhaiyalal Agrawal, Girish Kumar Parida, Gopinath Gnanasegaran

**Affiliations:** 1 Department of Nuclear Medicine, All India Institute of Medical Sciences, Bhubaneswar, Odisha 751019 India; 2 Department of Nuclear Medicine, Royal Free London NHS, London, UK

**Keywords:** ⁶⁸Ga-Pentixafor PET/CT, CXCR4 imaging, Primary aldosteronism, Aldosterone-producing adenoma, Adrenal venous sampling, Functional adrenal imaging, CYP11B2

## Abstract

Primary aldosteronism (PA) is the most common cause of secondary hypertension, where accurate subtype differentiation between unilateral aldosterone-producing adenoma (APA) and bilateral adrenal hyperplasia (BAH) determines therapeutic strategy. Adrenal venous sampling (AVS) remains the reference standard but is invasive, technically demanding, and not widely available. Recent advances in molecular imaging have introduced CXCR4-targeted positron emission tomography using ⁶⁸Ga-Pentixafor as a promising non-invasive tool for functional characterization and lateralization of aldosterone-producing lesions. CXCR4 is highly expressed in the zona glomerulosa and in aldosterone-producing adenomas, and it closely correlates with CYP11B2 (aldosterone synthase) expression. Across multiple clinical studies, ⁶⁸Ga-Pentixafor PET/CT has demonstrated high diagnostic accuracy, with reported sensitivities ranging from 74% to 98% and specificities from 84% to 100%, showing substantial concordance with AVS findings. In addition, PET-guided adrenalectomy yields biochemical and clinical outcomes comparable to AVS-guided management while avoiding procedural risks. By targeting CXCR4 expression, ⁶⁸Ga-Pentixafor PET/CT offers reliable lateralization with good agreement to AVS and strong clinicopathological correlation. Ongoing studies will further clarify its diagnostic role and integration into routine evaluation of primary aldosteronism.

## Introduction

Primary aldosteronism (PA) is the leading cause of secondary hypertension, constituting a distinct clinical entity that is specifically treatable and, in many cases, curable. It is estimated to contribute to approximately 5–10% of all hypertension cases. Compared with patients with essential hypertension, individuals with primary aldosteronism exhibit a substantially elevated risk of developing cerebrovascular and cardiovascular complications, including stroke, coronary artery disease, atrial fibrillation, and heart failure (Monticone et al. 2018). Therefore, the timely diagnosis and appropriate management of primary aldosteronism are crucial for improved long-term prognosis.

In PA, aldosterone secretion exceeds physiological needs and becomes relatively autonomous from its normal regulator, the renin–angiotensin II system. This results in enhanced sodium reabsorption through amiloride-sensitive epithelial sodium channels within the distal nephron, leading to arterial hypertension and suppression of renin–angiotensin activity. Excessive sodium reabsorption in the distal nephron enhances potassium and hydrogen ion secretion into the urine, which, if persistent, can lead to hypokalemia and metabolic alkalosis.

Pathologically, primary aldosteronism (PA) can occur in unilateral form, including aldosterone-producing adenoma (APA), aldosterone-producing micronodule (APN), and unilateral adrenocortical hyperplasia (UAH) and bilateral forms, such as idiopathic or bilateral adrenal hyperplasia (IHA/BAH) and, rarely, aldosterone-producing adrenocortical carcinoma (APACC).

## Diagnosis

High-risk hypertensive patients should be evaluated for primary aldosteronism regardless of potassium level, since normokalemic hypertension (~ 80%) is far more common than the hypokalemic variant.

Evaluation of suspected primary aldosteronism begins with simultaneous measurement of plasma aldosterone concentration (PAC) and either plasma renin activity (PRA) or direct renin concentration (DRC) (Adler et al. 2025).

- Renin is low or suppressed (hallmark of the diagnosis), PRA is ≤ 1 ng/mL/h or direct renin concentration (DRC) is ≤ 8.2 mU/L.
- The PAC is > 10 ng/dL (277 pmol/L).
- The aldosterone to renin ratio (PAC/PRA ratio) is > 20.

After aldosterone-suppression tests, such as the salt-loading test, saline infusion test, or fludrocortisone suppression test, and the captopril challenge test, a positive screening diagnosis can be confirmed.

## Role of subtyping in primary aldosteronism 

Once the diagnosis of primary aldosteronism (PA) is established, it is essential to differentiate between unilateral and bilateral forms of aldosterone excess. This distinction carries therapeutic implications. The two major forms are unilateral aldosterone-producing adenoma (APA) and bilateral adrenal hyperplasia (BAH), also referred to as idiopathic hyperaldosteronism (IHA). The bilateral form is more common (60–70%) than the unilateral.

**APA** accounts for approximately 35% of PA patients. Unilateral laparoscopic adrenalectomy is considered the treatment of choice unless the patient declines surgery or is surgically unfit, resulting in improvement in hypertension and biochemical parameters in most patients and often a cure (Adler et al. 2025). Clinically, individuals with APA tend to present at a younger age (commonly below 50 years) and demonstrate more pronounced hypertension, marked hypokalemia (serum potassium < 3.2 mEq/L), and significantly elevated plasma (> 25 ng/dL) and urinary (> 30 µg/24 h) aldosterone concentrations compared with those having bilateral disease (Young 2007).

**Idiopathic or bilateral adrenal hyperplasia (IHA/BAH)**, on the other hand, is a less severe form and generally managed medically with mineralocorticoid receptor antagonists (Adler et al. 2025).

In individuals for whom surgery is considered, adrenal lateralization is performed using computed tomography (CT) and adrenal venous sampling (AVS) before deciding on management (medical or surgical).

Adrenal computed tomography (CT) is the initial study to determine subtype and exclude adrenal carcinoma. CT has several limitations, as small adenomas may remain undetectable on CT imaging; conversely, lesions that appear as microadenomas on CT can, in fact, represent focal areas of adrenal hyperplasia, rendering unilateral adrenalectomy inappropriate. In addition, non-functioning unilateral adrenal macroadenomas are frequently encountered in individuals over 35 years of age, which cannot be reliably differentiated from functioning APAs using CT imaging alone. Several studies have proven low concordance (~ 40–50%) between CT and AVS.

In one meta-analysis of 25 studies involving 4669 patients, the analysis revealed a pooled sensitivity of 68% and specificity of 57% for CT/MRI in identifying unilateral PA (Zhou et al. 2020). Even in young patients (≤ 40 years), 21% would have undergone unnecessary adrenalectomy based on imaging results alone. In another systematic review of 38 studies, CT/MRI results did not agree with AVS results in 37.8% of patients (Kempers et al. 2009). A treatment decision based solely on CT findings may be considered in selected patients with a very high likelihood of unilateral disease, such as those with a > 1.0 cm unilateral adrenal adenoma, marked primary aldosteronism and hypokalemia.

## Adrenal vein sampling (AVS)

Adrenal venous sampling (AVS) is currently the gold standard for identifying the lateralization of aldosterone secretion (Rossitto et al. 2020).

AVS is usually performed with cosyntropin stimulation. Blood samples are collected from the adrenal veins and the inferior vena cava (IVC) below the renal veins. Successful adrenal venous sampling (AVS) is confirmed when the adrenal vein–to–inferior vena cava (IVC) cortisol ratio is more than 5:1. A cortisol-corrected aldosterone ratio, calculated as the aldosterone-to-cortisol ratio from the dominant (APA) side divided by that from the contralateral adrenal gland, greater than 4:1 is indicative of unilateral aldosterone hypersecretion, whereas a ratio of less than 3:1 suggests bilateral aldosterone hypersecretion (Adler et al. 2025).

Despite its diagnostic accuracy, AVS has some limitations, including invasiveness, high cost, scarce availability, need for an experienced interventional radiologist, technical challenges (with only a 50%–80% success rate for right adrenal vein cannulation), a lack of standardised protocols and occasional complications such as groin hematoma (Mulatero et al. 2010). It has driven efforts to develop reliable alternative non-invasive tools to identify functional lesions and lateralize aldosterone secretion.

## Functional imaging of adrenocortical tumors

(¹³¹I/¹²³I)-6β-iodomethyl-19-norcholesterol (NP-59), a cholesterol analogue taken up by adrenocortical cells via LDL receptor–mediated mechanisms, was the first radiotracer used for functional evaluation of adrenal incidentalomas. ^11^C-Metomidate (MTO) PET/CT, a specific adrenocortical tracer targeting 11β-hydroxylase (CYP11B1) and aldosterone synthase (CYP11B2), accurately distinguishes adrenocortical from noncortical lesions but remains limited by its short half-life, the need for on-site cyclotron production, and restricted availability (Wu et al. 2023). ¹⁸F-FDG PET/CT can help differentiate benign from malignant adrenal lesions; however, its specificity is limited. Physiologic uptake in normal adrenal tissue, uptake in non-functioning adenomas, and variable metabolic activity of functional nodules make it unreliable for functional characterization of adrenocortical lesions.

## Rationale for ^68^Ga-Pentixafor imaging in primary aldosteronism

Chemokines are a specialized subgroup of cytokines involved in physiological organ development and cell migration. The chemokine receptor 4 (CXCR4) and 7 (CXCR7) are G protein-coupled receptors localized on the cell membrane and activated through their shared ligand CXCL12 in multiple human cancers. They are involved in various intracellular signalling pathways, playing a key role in the tumor and tumor microenvironment (TME) by promoting cell migration, proliferation, survival and orchestrating recruitment of immune and stromal cells within the TME (Santagata et al. 2021). Because of high expression of CXCR4, particularly in the zona glomerulosa of the adrenal cortex, CXCR4 expression is markedly upregulated in aldosterone-producing adenomas (APAs) and cortisol-producing adenomas (CPAs), and also correlates strongly with CYP11B2 (aldosterone synthase) expression (Heinze et al. 2018). In contrast, its expression is either weak or absent in non-functioning adenomas (NFAs) and paragangliomas (Kaemmerer et al. 2017), underscoring its specificity for adrenocortical tumors and its potential as a molecular marker distinguishing functional from non-functional adrenal tumors. Several CXCR4-targeted radiotracers have been developed as synthetic analogues of its natural ligand, CXCL12, with ⁶⁸Ga Pentixafor being the most extensively studied and clinically validated. Most published studies have focused on the role of ⁶⁸Ga-Pentixafor PET/CT, demonstrating the robust diagnostic potential for the functional subtyping of primary aldosteronism (PA) (Table 1).

**Table 1:** Summary of studies evaluating the role of ^68^Ga-Pentixafor PET in the assessment of Primary Aldosteronism

Study	Year	Number of patients in each category	Study Design	Final diagnosis based on	Results (visual analysis)	Semi-quantitative Analysis	Conclusion
Ding et al. 2022	**2022**	64 (40 APA, 3 UAH, 8 IAH, 8 NFA, 3 CPA, 2 PCC)	Retrospective	Histopathological examination and follow-up	Sensitivity 97.8%, specificity 87.5%	SUVmax ≥ 7.1: sensitivity 90.0%, specificity 85.3%; LLR ≥ 2.5: sensitivity 95.5%, specificity 88.2%; LCR ≥ 2.4: sensitivity 88.6%, specificity 91.8%	^68^Ga-Pentixafor exhibited excellent sensitivity and specificity for the functional lateralization of adrenal disease.
Gao et al. 2022	2022	60 (39 APA, 11 IHA, 10 NFA)	Prospective	Postoperative histopathological findings	Sensitivity 93.0%, specificity 84.6%	SUVmax ≥ 8.95: sensitivity 76.7%, specificity 92.3%, accuracy 80.4%	At least 90% of functional nodules showed positive uptake on ^68^Ga-Pentixafor PET/CT. ^68^Ga-Pentixafor PET/CT can be considered a promising tool for surgical decision-making in patients with PA.
Zheng et al. 2023	2023	120 (66 APA, 33 IHA, 21 NFA)	Prospective	Histopathology, follow-up, AVS	Sensitivity 92.4%, specificity 94.4%, accuracy 93.3% 77 underwent surgery, and all PET-positive patients benefited (100% cure/improvement)	SUVmax ≥ 7.65: sensitivity 84.8%, specificity 90.7%, accuracy 87.5%; LAR ≥ 1.60: sensitivity 87.9%, specificity 88.9%, accuracy 88.3%; LLR ≥ 3.36: sensitivity 86.4%, specificity 94.4%, accuracy 90.0%	^68^Ga-Pentixafor PET/CT provides high accuracy in classifying PA and guiding clinical treatment decision-making.
Ding et al. 2024	**2024**	104 patients with PA **and adrenal micronodules (< 1 cm)**	Prospective	Post-surgical follow-up and AVS	Sensitivity 90.2%, specificity 72.7%, accuracy 86.5%; concordance with AVS 66.7% (36 patients)	SUVmax ≥ 4.55: sensitivity 84.8%, specificity 84.6%; LLR ≥ 2.17: sensitivity 76.1%, specificity 89.0%; LAR ≥ 1.90: sensitivity 84.8%, specificity 90.1%	68Ga-Pentixafor PET/CT holds promise in outperforming adrenal CT for the classification of micronodules in PA and can guide surgical management effectively.
Yin et al. 2024	2024	26 (19 UPA, 7 BPA)	Prospective, head-to-head comparison with AVS	Post-surgical follow-up and AVS	Sensitivity 89%, accuracy 92%; concordance with AVS 77%	SUVmax ≥ 5.71: sensitivity 78.95%, specificity 100%; LCR ≥ 1.39: sensitivity 89.47%, specificity 100%; LLR ≥ 3.05: sensitivity 94.74%, specificity 100%	^68^Ga-Pentixafor PET/CT is a reliable tool for PA subtype diagnosis, and it also identified additional cases of unilateral surgically curable PA for which AVS failed to perform classification.
Hu et al. 2023	2023	43 UPA 57 BPA	Prospective	AVS	Concordance with AVS 90%	Lateralization index LI = 1.65 at 10 min: sensitivity 77.0%, Specificity 100%; LI = 1.57 at 40 min: sensitivity 86.0%, specificity 91.0%	^68^Ga-Pentixafor PET/CT may be used to avoid invasive AVS in some patients with PA.
Peng et al. 2025	2025	95(83 UPA, 4 BPA, 8 NFA)	Prospective study evaluating ¹⁸F-AlF-NOTA-Pentixafor PET/CT	Post-surgical follow-up and AVS	Sensitivity 89.7%, specificity 94.1%; concordance with AVS 65%	SUVmax ≥ 5.45: sensitivity 79.5%, specificity 100%; LAR ≥ 1.43: sensitivity 83.3%, specificity 100%; LLR ≥ 3.20: sensitivity 80.8%, specificity 100%	The CXCR4-targeted 18 F-AlF-NOTA-Pentixafor PET/CT is a valuable non-invasive tool for diagnosing UPA, demonstrating high sensitivity and specificity.
Lu et al. 2025	2025	50 (27 APA, 5 NFA, 11 UHP, 7 IHA)	Prospective	Post-surgical follow-up, head-to-head comparison with AVS	Sensitivity 73.7%, specificity 100.0%, concordance with AVS was 96.2% in APA patients and 75.7% in all patients	SUVmax ≥ 11.95: sensitivity 57.89%, specificity 100.0%; SUVmax ≥ 5.85: sensitivity 81.6%, specificity 73.1%	^68^Ga-Pentixafor PET/CT imaging, as a non-invasive method, performs excellently in detecting APA and could act as an effective supplement to adrenal venous sampling in PA lateralization.
Zuo et al. 2025	2025	161 (90 UPA, 71 BPA)	Retrospective	Post-surgical follow-up and AVS	Diagnostic accuracy at 10 min: 75.2%, accuracy at 40 min: 76.4%	LCR = 1.95 at 10 min, sensitivity, specificity, and accuracy of 76.0%, 91.3%, and 83.3% LLR = 4.79 at 40 min, sensitivity 90.0%, specificity 66.7%.	^68^Ga-Pentixafor PET/CT exhibits robust diagnostic efficacy in PA lateralization as well as holds promise as an imaging marker for predicting the presence of the KCNJ5 mutation in PA patients
Wen-Cai Zheng et al. 2025	2025	90	Prospective study to predict the surgical outcomes compared to AVS	Post-surgical follow-up			Pentixafor PET/CT had similar accuracy to AVS for predicting post-surgical biochemical and clinical success.
Lu Tan et al. 2025	2025	89 unilateral PA patients (23 PET group and 66 AVS group)	Retrospective cohort study, head-to-head comparison with AVS in guiding surgical management	Post-surgical follow-up			Postoperative clinical and biochemical outcomes were comparable between AVS- and PET-guided groups.
Zheng et al. 2025	2025	197 PA patients (137 PET group and 60 AVS group)	Prospective cohort study, head-to-head comparison with AVS in guiding surgical management	Post-surgical follow-up		PET: SUVmax ≥ 5.5: sensitivity 82.8%, specificity 92.6%	^68^Ga-Pentixafor PET/CT could effectively guide the surgical management for PA, achieving favourable clinical and biochemical outcomes not inferior to AVS.

## Diagnostic performance of Pentixafor PET in subtyping primary aldosteronism

The initial proof-of-concept study of ⁶⁸Ga-Pentixafor PET/CT in aldosterone-producing adenomas was conducted by Heinze et al. who investigated nine patients and reported sensitivities and specificities of 88% and 87%, respectively. One APA nodule showing uptake below the cutoff SUVmax, it measured 6 mm (Heinze et al. 2018). Subsequently, Ding et al. retrospectively evaluated 64 patients with adrenal masses and found sensitivities and specificities of 97.8% and 87.5%, respectively. The two pheochromocytoma lesions had lower uptake than the normal adrenal glands (Ding et al. 2022).

Lu et al. prospectively studied 50 patients with confirmed primary aldosteronism to determine the diagnostic efficiency of ⁶⁸Ga-Pentixafor PET/CT relative to AVS. Using an SUVmax cut-off of 11.95 in aldosterone-producing adenomas yielded 74.1% sensitivity and 100% specificity. Concordance between PET/CT and AVS reached 96.2% for APA and 75.7% overall (Lu et al. 2025). Furthermore, in a cohort of 90 patients with primary aldosteronism, Hu et al. demonstrated a 90% concordance between ⁶⁸Ga-Pentixafor PET/CT and adrenal venous sampling (AVS) (Hu et al. 2023). Notably, in the subgroup of patients with unilateral adrenal nodules larger than 1 cm on CT (*n* = 40), the concordance rate reached 100%. Good diagnostic performance has been observed for both micro and macronodular adrenal lesions across various studies, although sensitivity drops for lesions less than 1 cm due to limited resolution. Nevertheless, ⁶⁸Ga-Pentixafor PET/CT consistently outperforms morphologic CT for subcentimeter lesions. **Ding et al.** prospectively evaluated 104 patients with primary aldosteronism and adrenal micronodules (< 1 cm). PET/CT achieved a sensitivity of 90.2%, specificity of 72.7%, and overall accuracy of 86.5% for detecting unilateral disease (Ding et al. 2024). A recent diagnostic meta-analysis pooling seven studies (603 patients) reported that ^68^Ga-Pentixafor PET/CT has a pooled sensitivity of 80% (95% CI 70–87%), specificity of 91% (95% CI 86–94%) and an AUC of 0.91 for identifying unilateral primary aldosteronism, with PET/CT showing agreement with AVS in ~ 79% of paired cases — findings that support a substantial rule-in value for PET positivity (positive post-test probability rising to ~ 96% from pre-test probability of 75%) (Gege et al. 2025).

Visual analysis performed comparably to, or slightly better than, semiquantitative SUV-based assessment for initial lesion detection and lateralization of PA, with sensitivity ranging from 74% to 98% and specificity from 84% to 100%. A lesion is generally considered visually positive when uptake is more than that of the contralateral adrenal gland. Semiquantitative metrics are reproducible, complementary to visual analysis, and improve specificity; reported SUVmax cut-offs for differentiation across studies range from 4.5 to 11.95. Lesion-to-contralateral adrenal (LCR/LI) and lesion-to-liver (LLR) ratios are widely used to assess lateralization, with threshold values generally ranging from 1.4 to 3.4 to achieve high specificity (Zhang et al. 2025).

**Fig. 1:**
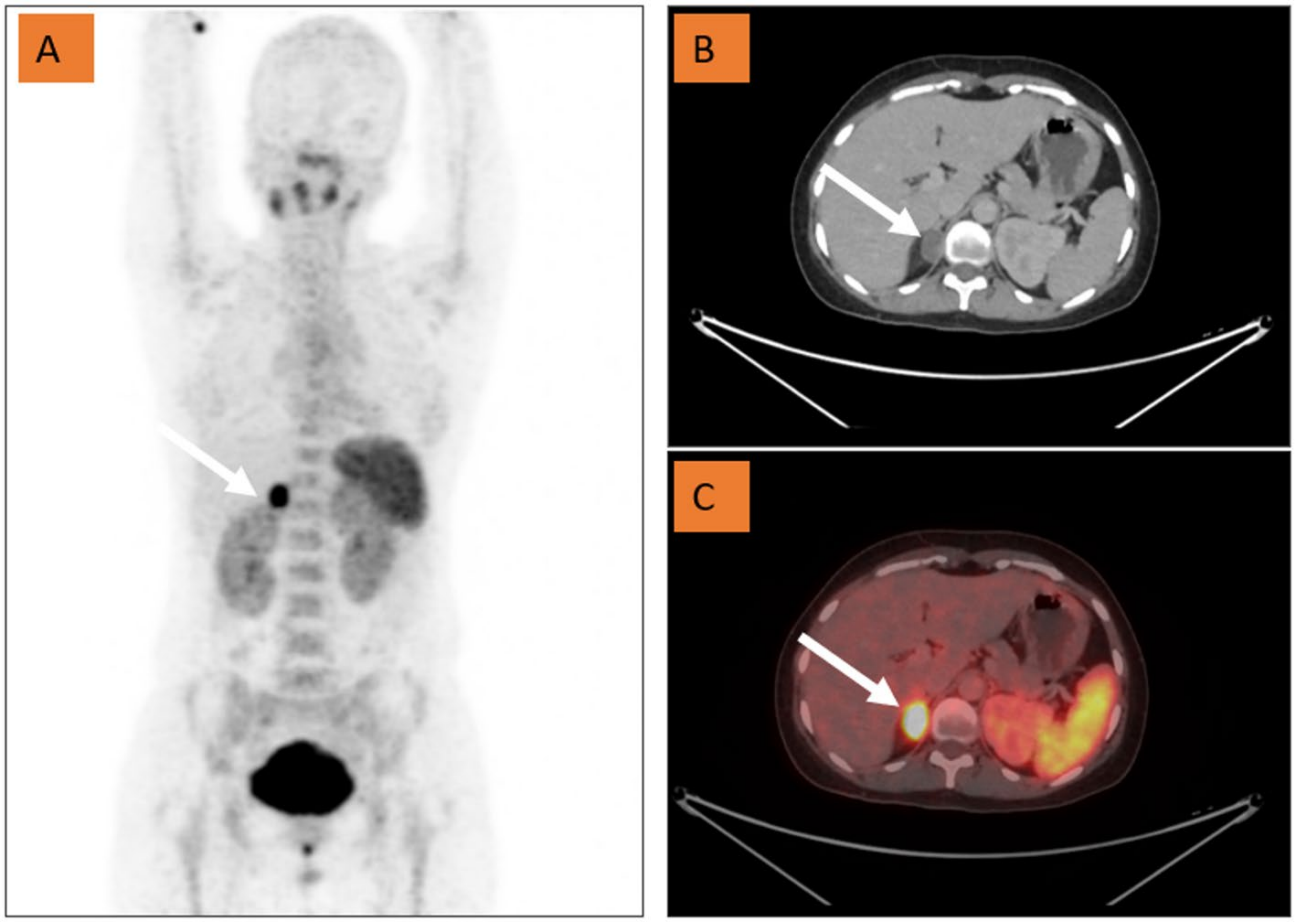
Ga-68 Pentixafor PET-CT (**A**) Maximum Intensity Projection PET image showing intense uptake in the right suprarenal region. (**B**-**C**) Axial CT and fused PET-CT images show increased tracer uptake in the hypodense nodule in the right adrenal gland (arrow), suggestive of a unilateral aldosterone producing adenoma

The intensity of ⁶⁸Ga-Pentixafor uptake, measured as the maximum standardized uptake value (SUVmax), demonstrates a clear gradient across adrenal subtypes. Median or mean SUVmax values are consistently highest in aldosterone-producing adenomas (APA) [Fig. 1], low to intermediate in idiopathic or bilateral adrenal hyperplasia (IHA/BAH), and lowest or absent in non-functioning adenomas (NFA) and normal adrenal glands.

Across studies, APA lesions typically show median SUVmax values of 9-14.5, IHA or bilateral lesions display low to moderate uptake (~ 4–6), and NFAs or contralateral normal glands usually remain near background levels (~ 2–4). This stepwise difference is statistically significant (*P* < 0.001 in all studies) and parallels the immunohistochemical expression of CYP11B2 and CXCR4, indicating true tracer specificity for aldosterone-producing tissue. Moreover, SUVmax values in patients achieving postoperative biochemical cure are significantly higher than those in patients with partial or no improvement, supporting its prognostic relevance. Higher uptake correlates strongly with unilateral disease, lower serum potassium levels, and larger lesion diameters. In contrast, uptake in bilateral IHA lesions is generally symmetrical and of lower intensity, reflecting diffuse rather than focal aldosterone production.

## Impact of ⁶⁸Ga-Pentixafor PET/CT on patient management and success after PET-guided surgery management

The value of ⁶⁸Ga-Pentixafor PET/CT in primary aldosteronism extends beyond diagnosis to the optimization of surgical decision-making. Surgical outcomes are classified according to PASO (Primary Aldosteronism Surgical Outcome) criteria. Multiple prospective studies have demonstrated that surgical outcomes guided by 68Ga-Pentixafor PET/CT are non-inferior to those guided by adrenal venous sampling (AVS). In a large single-centre prospective cohort of 197 patients with primary aldosteronism, surgery guided by ^68^Ga-Pentixafor PET/CT (*n* = 137) was compared with surgery guided by AVS findings (*n* = 60), with a median follow-up of 27 months. Complete clinical and biochemical success was achieved in 51.5% and 82.1% of PET-guided cases versus 35.1% and 75.4% with AVS (*P* > 0.1). Overall benefit rates (complete and partial success) were 95.5% for PET-guided and 93% for AVS-guided surgery, proving the non-inferiority of PET/CT-guided management. In patients with unilateral PET-positive and contralateral PET-negative findings, postoperative benefit reached **100%** (Zheng et al. 2025b).

In the study by Lu et al., two separate cohorts of patients who underwent adrenalectomy and had unilateral PA (66 in the AVS group and 23 in the CXCR4 group) were analysed. Among these, 74 (83.2%) achieved biochemical success (80.3% in the AVS group vs. 91.3% in the CXCR4 group), while 75 (84.3%) showed clinical improvement (81.8% vs. 91.3%, respectively). Notably, the overall surgical cure rate was 60.7% (59.1% in the AVS group vs. 65.2% in the CXCR4 group), with no statistically significant difference between the two approaches (Tan et al. 2025). In the study by Wen-Cai Zheng et al. postoperative outcomes did not differ significantly between patients who underwent partial adrenalectomy (*n* = 45) and those who underwent total adrenalectomy (*n* = 45). They found that the accuracy of ⁶⁸Pentixafor PET/CT in correctly predicting complete biochemical success and complete clinical success was 77.78% and 67.78%, respectively, while for AVS, the corresponding accuracies were 73.33% and 54.44%, respectively (Zheng et al. 2025a). Notably, when unilateral intense uptake is present with a contralateral-negative gland, postoperative benefit rates approached 100%, demonstrating that high SUVmax or lateralization ratio can predict curative surgery with exceptional accuracy.

## Dosimetry, administration, and biodistribution of ^68^Ga-Pentixafor

^68^Ga-Pentixafor has a favourable safety and dosimetry profile, as well as well-defined radiochemistry and administration procedures in human imaging. The typical injected activity is around 150 MBq (150 ± 50 MBq), producing an effective whole-body dose of ~ 2.3 mSv for 150 MBq. The organs receiving the highest absorbed doses were the urinary bladder wall (12.2 mGy), spleen (8.1 mGy), kidneys (5.3 mGy), and myocardial wall (4.0 mGy). For ^68^Ga-Pentixafor, the absorbed doses to the liver, kidney, and spleen are much lower than for the other 68Ga-labelled radiopharmaceuticals. Excretion occurs rapidly via the kidneys, and only about 4–8% of the injected activity remains in the body at 4 h. Following tracer injection, the optimal lesion-to-background contrast is typically achieved after 30 min, and image acquisition is performed between 30- and 60-minutes post-injection (Herrmann et al. 2015). Some authors have used dual acquisitions; Hu et al. acquired images at 10 and 40 min after injection and observed that the 10-minute acquisition provided superior diagnostic accuracy for identifying the dominant adrenal gland in primary aldosteronism (Hu et al. 2023).

## Conclusion

Accurate detection of functional adrenal lesions and correct lateralization of aldosterone production are vital for determining management strategies in primary aldosteronism, as these factors directly impact postoperative outcomes, particularly in patients presenting with small or bilateral adrenal lesions. ^68^Ga-Pentixafor PET/CT represents a significant advancement in the non-invasive evaluation of primary aldosteronism. By enabling functional imaging of CXCR4 expression, a chemokine receptor markedly upregulated in aldosterone-producing adenomas, it allows accurate lesion localization, reliable lateralization of disease, and improved surgical planning, thereby improving patient selection for adrenalectomy.

It has high diagnostic accuracy and good concordance with adrenal venous sampling (AVS) for disease lateralization. It surmounts several inherent AVS limitations, including technical complexity, invasiveness, operator dependency, and limited availability across centres. The studies have also demonstrated high accuracy in detecting small adrenal lesions that may not be well characterized on conventional imaging.

By effectively imaging cortisol-producing adenomas, it also distinguishes functional from non-functional adrenal nodules with high confidence. Given its strengths, ⁶⁸Ga-Pentixafor PET/CT has the potential to serve as a viable alternative or adjunct to AVS, particularly in institutions without facilities for venous sampling.

In addition, Technetium-99 m–labelled CXCR4 analogue, [⁹⁹ᵐTc]-Pentixatec, has been investigated in preliminary studies and may offer a more accessible and cost-effective SPECT-based option in the future (Enke et al. 2024). Although encouraging, current findings stem largely from single-country and single-centre studies. Additional large, multicentre, prospective trials are needed to validate its diagnostic performance further, quantify its impact on clinical decision-making, and assess its ability to predict postoperative biochemical and clinical outcomes, thereby making it an accepted modality for subtyping primary aldosteronism.

## Data Availability

Not applicable.

## Abbreviations

PA: primary aldosteronism
APA: aldosterone-producing adenoma
IAH: idiopathic aldosterone hypersecretion
NFA: non-functioning adenoma
CPA: cortisol-producingadenoma
PCC: pheochromocytoma
PET/CT: positron emission tomography/computedtomography
SUVmax: maximum standardized uptake value
AVS: adrenal venous sampling
LCR: lesion-to-contralateral ratio
LLR: lesion-to-liver ratio
LAR: lesion-to-adrenal ratio
UPA: unilateral primary aldosteronism
BPA: bilateral primary aldosteronism
BAH: bilateral adrenalhyperplasia
CXCR4: C-X-C motif chemokine receptor 4
AUC: area under the curve
CI: confidence interval
